# Involvement of Ceramide Metabolism in Cerebral Ischemia

**DOI:** 10.3389/fmolb.2022.864618

**Published:** 2022-04-20

**Authors:** Alberto Ouro, Clara Correa-Paz, Elena Maqueda, Antía Custodia, Marta Aramburu-Núñez, Daniel Romaus-Sanjurjo, Adrián Posado-Fernández, María Candamo-Lourido, Maria Luz Alonso-Alonso, Pablo Hervella, Ramón Iglesias-Rey, José Castillo, Francisco Campos, Tomás Sobrino

**Affiliations:** ^1^ NeuroAging Group (NEURAL), Clinical Neurosciences Research Laboratory (LINC), Health Research Institute of Santiago de Compostela (IDIS), Santiago de Compostela, Spain; ^2^ Translational Stroke Laboratory Group (TREAT), Clinical Neurosciences Research Laboratory (LINC), Health Research Institute of Santiago de Compostela (IDIS), Santiago de Compostela, Spain; ^3^ Neuroimaging and Biotechnology Laboratory (NOBEL), Clinical Neurosciences Research Laboratory (LINC), Health Research Institute of Santiago de Compostela (IDIS), Santiago de Compostela, Spain

**Keywords:** ceramide, cerebral ischemia, metabolism, hypoxia, inflammation, stroke

## Abstract

Ischemic stroke, caused by the interruption of blood flow to the brain and subsequent neuronal death, represents one of the main causes of disability in worldwide. Although reperfusion therapies have shown efficacy in a limited number of patients with acute ischemic stroke, neuroprotective drugs and recovery strategies have been widely assessed, but none of them have been successful in clinical practice. Therefore, the search for new therapeutic approaches is still necessary. Sphingolipids consist of a family of lipidic molecules with both structural and cell signaling functions. Regulation of sphingolipid metabolism is crucial for cell fate and homeostasis in the body. Different works have emphasized the implication of its metabolism in different pathologies, such as diabetes, cancer, neurodegeneration, or atherosclerosis. Other studies have shown its implication in the risk of suffering a stroke and its progression. This review will highlight the implications of sphingolipid metabolism enzymes in acute ischemic stroke.

## Introduction

Stroke is a cerebrovascular disease resulting from a disturbance in normal cerebral blood flow (CBF), which causes transient or permanent deficits in the function, becoming one of the leading causes of disability in developed countries. The disturbance of normal CBF induces metabolic and cellular changes that can lead to cell death and disruption of the nervous system (Powers et al., 2019). Stroke can be classified into two types: hemorrhagic and ischemic stroke. A hemorrhagic stroke is due to a blood vessel rupture, and represents up to 15% of all the cases, and is associated with high mortality risk. In contrast, an ischemic stroke is caused by an obstruction of a cerebral vessel, and constitutes approximately 85% of all strokes (Campbell et al., 2019).

**FIGURE 1:**
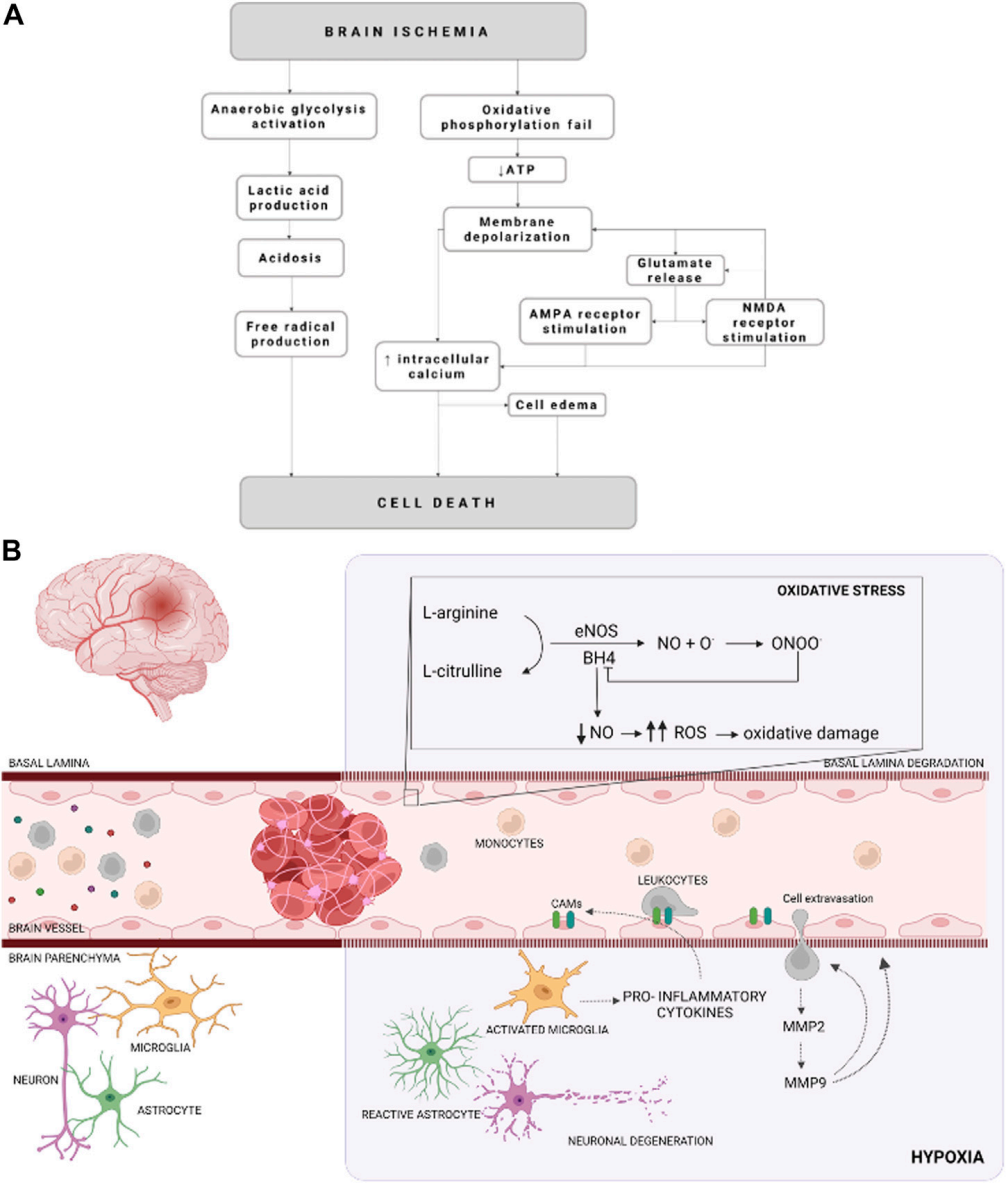
Schematic representation of cellular mechanisms during an ischemic stroke. **(A)** Schematization of the molecular events that occurred due to an ischemic event. **(B)** Illustration of cellular and molecular events on cerebral ischemia. Cellular adhesion molecules (CAMs), reactive oxygen species (ROS), endothelial nitric oxide synthase (eNOS), nitric oxide (NO), metalloproteinase (MMP), tetrahidrobiopterine (BH4), oxygen radicals (O^.^), and peroxynitrite (ONOO^−^) are represented by their acronyms.

Acute ischemic stroke is a dynamic process in which multiple molecular and cellular processes are involved (defined as an ischemic cascade), which starts immediately after the ischemic insult and changes even weeks and months later (Agulla et al., 2013; Brea et al., 2015). Based on the blood perfusion level and metabolic activity in the infarct brain, two regions have been widely described: the ischemic core and the penumbra area (Ermine et al., 2021). The ischemic core is the most deeply affected region by the lack of blood flow and constitutes irreversible brain-injured within a few minutes from stroke onset. The penumbra area is a vulnerable, hypoperfused, and metabolically compromised brain region that can be recovered if the blood flow supply is re-established during the first few hours after a stroke. The ischemic penumbra area remains also the target of multiple protective drugs aimed to reduce or block the progression of cell death (Castellanos et al., 2006; Ramos-Cabrer et al., 2011; Silva-Candal et al., 2017; Correa-Paz et al., 2021). After the onset of cerebral ischemia, a sequence of pathophysiological processes occurs, which are comprised of oxidative stress, inflammation, breakdown of the blood-brain barrier (BBB), calcium overload, excitotoxicity, and autophagy dysfunction (Rodríguez-Yáñez et al., 2011; He et al., 2021) (Figure 1).

The therapeutic approaches after stroke may be classified into four groups: monitoring of cerebral homeostasis, reperfusion, neuroprotection and neurorepair strategies (Díez-Tejedor and Fuentes, 2005; Hurtado et al., 2006). Mechanical thrombectomy and thrombolysis are the reperfusion strategies, which are based on the restoration of the CBF. Neurorepair involves the restoration of brain function by regenerating the damaged cerebral tissue or establishing alternative neural pathways or synapses (Kim J. H. et al., 2021; Romaus-Sanjurjo et al., 2021). Neuroprotection has been focused on reducing cell death after an ischemic event intervening in the ischemic cascade mechanisms such as inflammation, oxidative stress, endothelial damage, or excitotoxicity (Chamorro et al., 2016). Despite that several compounds have been tried in preclinical and clinical studies, unfortunately so far, there have not yet available neuroprotective drug for stroke patients (Chamorro et al., 2021; Correa-Paz et al., 2021, 2022). For all these reasons, it is imperative to search for new targets that constitute new therapeutic options for stroke.

Brain is enriched in lipids that are grouped as sphingolipids, glycerophospholipids, and cholesterol; presented almost at equal ratios. Importantly, both cholesterol and sphingolipids are the principal components of lipid rafts (Hussain et al., 2019). Lipid rafts are considered as dynamic domains of 10–200 nm involved in compartmentalization of cellular processes, stabilization of protein that interact with the membrane, cell-cell interaction and synaptic transmission, among others (Olsen and Færgeman, 2017). Exosomes are membrane-bound extracellular vesicles of 30–100 nm of diameter released from cells to the extracellular space. Previously, it was believed that these vesicles were cellular debris. However, in recent years it has been shown that exosomes serve as communications between cells by carrying essential components such as nucleic acids, proteins, lipids and other cellular components. Interestingly, it has been shown that the association between different molecules with lipid rafts facilitates the release of exosomes [reviewed by Li et al. (2018)]. It should be noted that changes in the lipid composition of the membrane, as well as of the lipid rafts, are implicated in the development and/or progression of diseases such as Alzheimer’s, Parkinson’s, different types of cancer or autoimmune diseases (Fabelo et al., 2011; Bodas et al., 2015; Loewith et al., 2019; Xue et al., 2019).

Sphingolipids were discovered as structural molecules, being the main components of cell membranes, as mentioned. However, sphingolipids have been shown to be pleiotropic molecules involved in cell regulation of several functions (Gangoiti et al., 2010; Summers et al., 2019). These molecules can regulate multiple pathways implicated in the control of cell functions like proliferation, apoptosis, autophagy, inflammation, angiogenesis, senescence, or polarization (Hannun and Obeid, 2008; Arana et al., 2010; Gangoiti et al., 2010; Ouro et al., 2012; Gomez-Larrauri et al., 2020; Quinville et al., 2021). Moreover, the dysregulation of sphingolipids metabolism leads to loss of cellular and organism homeostasis as evidenced by its involvement in different diseases such as cancer, diabetes, neurodegeneration and cardiovascular diseases (Iqbal et al., 2017; Gomez-Larrauri et al., 2020, 2021a; Custodia et al., 2021).

Ceramide (Cer) is considered the central/key molecule in sphingolipid metabolism. Cer is composed of sphingoid base (such as sphingosine or sphinganine) linked to a fatty acid that differs in chain length from 14 to 26 carbons. Interestingly, Cer is highly expressed in neurons, modulating neuronal signaling, synaptic transmission, cell metabolism, neuron-glia interaction, and cell survival (Schultz and Larsson, 2004; Gulbins et al., 2016; Cruciani-Guglielmacci et al., 2017). In addition, intracellular accumulation of Cer has been observed to be critical to induce neurodegeneration (Jana et al., 2009). Different studies have observed the relationship between the metabolism of Cer and the progression of cerebral ischemia, in both *in vitro* hypoxic experiments and in preclinical or clinical studies (Kang et al., 2010; Chao et al., 2019; Yusuf et al., 2019; Gui et al., 2020; Xia et al., 2020; Lee et al., 2021). Increased ceramide levels have been associated with reperfusion after an ischemic event (Yusuf et al., 2019). Cer is considered a pro-apoptotic molecule since the accumulation of intracellular natural Cer and treatment with short-chain Cer analogs promote the activation of the apoptotic cascade (de Palma and Perrotta, 2017; Gomez-Larrauri et al., 2021a). In addition, high levels of Cer have been observed in the serum of stroke patients with poor outcomes (Gui et al., 2020; Custodia et al., 2021; Gaggini et al., 2021). Furthermore, high levels of long-chain Cer have been associated with BBB disruption (Vutukuri et al., 2018; Bekhite et al., 2021). Moreover, elevated plasma levels of Cer were recently observed from stroke patients with large artery atherosclerosis, and cerebral small vessel disease (You et al., 2020).

The quantification of sphingolipids is mainly based on chromatographic techniques. Recently immunoassay techniques by ELISA have been also developed for their detection, such as sphingosine 1-phosphate (S1P) (Halilbasic et al., 2020). Formerly, the majority technique for the separation of sphingolipids and their quantification was based on the labeling of palmitic acid with radioactive tritium and the subsequent separation of sphingolipids in thin-layer chromatography (TLC) or enzymatic fluorescence labeling techniques. However, with the development of high-performance liquid chromatography (HPLC) with state-of-the-art tandem mass spectrometry (MS/MS) techniques, it has been possible to determine concentrations of the different sphingolipid species in biological samples without the need for radiolabelling. In recent years, electrospray ionization mass spectrometry approaches to measuring sphingolipids (sphingolipidomics) in plasma have revealed their possible use as vascular risk biomarkers in diabetes and cardiovascular diseases (Gaggini et al., 2021; Tippetts et al., 2021). Moreover, sphingolipidomics also showed its ability to study sphingolipids as potential biomarkers associated with neurodegenerative processes (Ayub et al., 2021), demonstrating the involvement of sphingolipids in their underlying molecular mechanisms. Specifically, recent works on cerebral ischemia showed an alteration of the sphingolipid profile, both in pre-clinical (Chao et al., 2019) and clinical studies (Sun et al., 2017; Gui et al., 2020; Lee et al., 2021). Sun and others detected elevated levels of sphinganine and some phospholipids in serum samples (Sun et al., 2017). Subsequently, Gui and co-workers demonstrated an association between the severity of the brain injury and increased levels of Cer (Gui et al., 2020). Later, Lee et al., also demonstrated the viability of Cer as a blood biomarker for the progression of patients after a stroke showing that control patients had higher levels of sphingosine 1-phosphate and very-long-chain Cer than patients with ischemic stroke. However, they reported an increase in long-chain Cer levels in ischemic stroke patients in correlation with the severity of the disease (Lee et al., 2021). The reduction of S1P levels was also observed in acute ischemic stroke patients compared with healthy subjects (Liu et al., 2020). It is well known that S1P is highly involved in inflammatory processes, being the most studied sphingolipid so far. However, this review is focused on the cellular processes that occur in ischemic stroke and their relation with the metabolism of Cer, since the role of S1P in ischemia has already been discussed in different recent works (Gaire and Choi, 2021; Lu et al., 2021).

## Cellular Mechanisms Involved in Ischemia

The reduction of the CBF (oligemia), and consequently the oxygen depletion (hypoxia), affects neuronal and glial function besides vascular alterations and inflammation. All of these molecular and cellular events, known as ischemic cascade, derived from lack of CBF lead to cell death. The ischemic cascade involves the energetic failure, ionic imbalance, excitotoxic glutamate efflux, loss of metabolic function with increased acidosis, oxidative stress, activation of pro-inflammatory signals and BBB disruption (Sandoval and Witt, 2008; Liu et al., 2012) (Figure 1).

Energetic failure begins due to the reduction of oxygen and glucose supply, leading to lower intracellular concentrations of ATP. Furthermore, ATP reduction compromises the ion pumps activity and the maintenance of electrical gradients (Sandoval and Witt, 2008; Majid, 2014). Hence, the pumps fail and Na^+^ is accumulated in the cytoplasm, promoting a cellular depolarization, which in turn facilitated the Cl^−^ and water uptake. In addition, Na^+^/Ca^2+^ exchanger is blocked and Ca^2+^ is also accumulated in the cytosol. Moreover, cell depolarization also alters neurotransmitters release, promoting glutamate excitotoxicity, which activates N-methyl-D-aspartate receptors (NMDARs), leading to an increase in repolarization. Since glutamate is the most abundant excitatory neurotransmitter in the brain, is the most released under ischemic conditions. In addition, there is an interruption of the oxidative phosphorylation process by the mitochondria respiratory chain, elevating superoxide anions (O^2-^) levels, which are converted to other reactive oxygen species (ROS) due to the acidic environment. ROS overproduction provokes mitochondrial depolarization and reduces ATP generation (Yang J. L. et al., 2018; He et al., 2020). It is well known that mitochondrial function is essential in the excitability and survival of the neuronal cells and serves as apoptotic regulators. Therefore, its dysfunction has been associated with the pathogenesis of neurodegenerative diseases and ischemic stroke (Yang J. L. et al., 2018; He et al., 2020).

In the early stages after ischemia ROS are not only produced by the mitochondrial depolarization, but also due to the activity of neuronal nitric oxide (NO) synthase (nNOS). NO is synthesized in the endothelial cells, neurons, glial cells, and macrophages by the activity of NOS (Haley and Lawrence, 2017; Janaszak-Jasiecka et al., 2021). So far, there are three isoforms described, neuronal NOS (nNOS) localized mainly in the nervous system cell; induced NOS (iNOS) which expression is induced in various cell types by pro-inflammatory cytokines; and endothelial NOS (eNOS), which is almost expressed exclusively in endothelial cells (Janaszak-Jasiecka et al., 2021). eNOS constitutively synthesizes NO by endothelial cells (Knottnerus et al., 2009; Roquer et al., 2009). This isoform requires the presence of tetrahidrobiopterine (BH4), as a co-factor (Verma et al., 2003). Interestingly, BH4 deficiency provokes the eNOS uncoupling, leading to the production of ROS, such as superoxide or hydrogen peroxide (Verma and Anderson, 2002; Verma et al., 2003; Vanhoutte et al., 2017). In addition, this excess of ROS limits NO bioavailability, causing in turn eNOS uncoupling (Janaszak-Jasiecka et al., 2021).

Furthermore, iNOS represents a major cytotoxic molecule since produces a large amount of NO, since it concerns mitochondrial electron transport (Kanwar et al., 2009; Förstermann and Sessa, 2012) and causes glutamate release and excitotoxicity, which synergizes with hypoxia to induce neuronal death (Kanwar et al., 2009; Brown and Neher, 2010; Förstermann and Sessa, 2012). There are a series of complex molecular events, triggered by ischemic stroke, as genes activation and expression, such as hypoxia-inducible factor 1 (HIF-1), and nuclear factor-kappa B (NF-κB). (Wierońska et al., 2021). These factors, in turn, activate some cytokines, such as tumor necrosis factor (TNF-α), interleukins (IL), and platelet-activating factor, which are related to the upregulation of cellular adhesion molecules (CAMs), that are expressed on leukocytes and endothelial cells surface in response to endothelial dysfunction. There are 3 major classes: the selectins (P-selectin, L-selectin, E-selectin), the β-integrins (CD11/CD18), and some immunoglobins, such as intercellular adhesion molecule 1 (ICAM-1), vascular cell adhesion molecule 1 (VCAM-1) or platelet endothelial cell adhesion molecule 1 (PECAM-1) (Verma and Anderson, 2002; Yilmaz and Granger, 2008; Roquer et al., 2009; Theofilis et al., 2021). Thus, CAMs mediates the inflammatory and pro-coagulant effects of endothelial cells (Theofilis et al., 2021).

The activation and upregulation of cytokines and CAMs, respectively, are related to ischemic stroke-derived inflammation, which starts with microglia activation and macrophages and neutrophils infiltration through the disrupted BBB. In turn, activated microglia release pro-inflammatory cytokines like TNF-⍺ and IL-6 (Castillo et al., 2003; Clausen et al., 2008; Lambertsen et al., 2012, 2019; Anrather and Iadecola, 2016), which induces CAMs expression triggering leukocyte recruitment and migration to the brain parenchyma, increasing the brain lesion (Zaremba et al., 2001; Sandoval and Witt, 2008; Weiss et al., 2009; Jin et al., 2013; Theofilis et al., 2021).

Another event in ischemic stroke, due to inflammation is endothelium dysfunction, referred as a reduction of the barrier tightness and an increase in leakiness, while the vessels remain largely intact (Andjelkovic et al., 2019; Bernstein et al., 2020). The brain endothelium takes part in a complex multicellular structure, the BBB, that selectively allow or restrict the passage of substances between these compartments and acts as the interface between the blood circulation and the central nervous system (CNS) (Nag, 2003; Brown et al., 2007; Daneman, 2012; Obermeier et al., 2013, 2016). The BBB maintains an environment that allows neurons to function properly by tightly controlling the passage of molecules and ions, instantaneously delivering nutrients and oxygen according to current neuronal needs, and protecting the brain from toxins and pathogens (Brown et al., 2007; Obermeier et al., 2013, 2016). Besides the increase of permeability, endothelium suffers a pro-inflammatory response due to oxidative stress, among other agents (Hassan et al., 2003; Blanco et al., 2005), known as activation, which is also related to pro-inflammatory cytokines release and CAMs expression (Petty and Lo, 2002; Sandoval and Witt, 2008). Once again, leukocytes are recruited to the activated endothelium, and the infiltration into the brain parenchyma is induced (Blanco et al., 2005; Jin et al., 2010; Gorina et al., 2014; Majid, 2014). In addition, activated endothelium promotes a switch in microglia phenotype from homeostatic to activated contributing to neuroinflammation by an increase in the production and release of ROS among others (Zhang, 2019). Furthermore, activated leukocytes induce basal lamina degradation by metalloproteinase (MMP) release, such as MMP-2 and MMP-9. In addition, MMP-9 release by leukocytes transmigrated to the ischemic brain promotes further neutrophil recruitment to the same site in a positive feedback manner and causes extensive BBB breakdown (Blanco et al., 2005; Brouns et al., 2011). Moreover, when the ischemic stroke occurs MMP-2 expression is increased MMP-9 expression is induced. Besides the leukocytes release, MMP-9 is also secreted by neurons, glial cells, and endothelial cells in early BBB disruption, due to oxidative stress (Brouns et al., 2011). In addition, MMP-9 is spatially and temporally correlated with loss of BBB integrity and is related to the formation of vasogenic edema and hemorrhagic transformation (Brouns et al., 2011), which is also due to ion transporter dysfunction at the BBB level, which particularly increased brain Na^+^ uptake (Jiang et al., 2018).

## Stroke and Ceramide Metabolism

So far, three main pathways of Cer production have been described, the *de novo*, the salvage, and the sphingomyelinase (SMase) pathway. In total, more than 30 enzymes involved in its production are described. It should be pointed that the pathways are highly regulated and can be altered by cellular stress signals, such as ROS or inflammation. It is well known that intracellular sphingolipid concentrations determine the final cell fate, for example, an increase in Cer levels with a decrease in the levels of its antagonist molecule ceramide 1-phosphate (C1P) lead to cell death (Gomez-Larrauri et al., 2021a). The phosphorylation of ceramide to produce C1P is carried out by an enzyme called Cer kinase (CERK); while its dephosphorylation to produce Cer occurs by the action of C1P phosphatase. These enzymes are not found in the main Cer formation pathways, named above. Of interest, C1P is highly involved in processes related to stroke such as cell migration (Granado et al., 2009b; Kim et al., 2012; Arana et al., 2013; Vera et al., 2021), invasion involving MMPs (Ordoñez et al., 2016a), cell proliferation through NO (Gangoiti et al., 2008) or vascular endothelial growth factor (VEGF) (Ouro et al., 2017), cell survival (Gómez-Muñoz et al., 2010; Gomez-Larrauri et al., 2021b), metabolism (Ouro et al., 2013), and inflammation (Nakamura et al., 2006; Gomez-Muñoz et al., 2013; Baudiß et al., 2016). What makes C1P also an interesting target for the study of its involvement in stroke. Although, this review focuses on the metabolism of Cer by the 3 main pathways; alternative, elegant reviews that address the subject of C1P in inflammatory processes can be studied (Lamour and Chalfant, 2008; Hinkovska-Galcheva and Shayman, 2010; Ouro et al., 2012; Hoeferlin et al., 2013; Gomez-Muñoz et al., 2016; Hait and Maiti, 2017).

### *de Novo* Pathway

**FIGURE 2:**
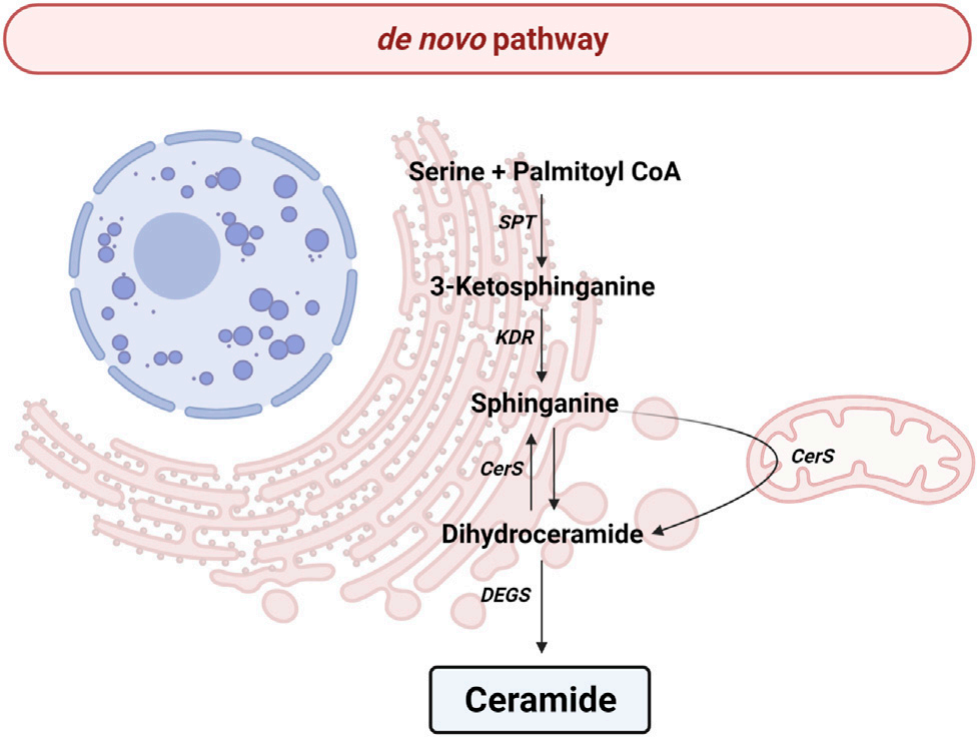
*de novo* pathway. Solid arrows represent single reactions. Serine palmitoyltransferase (SPT), 3-keto-dihydrosphingosine reductase (KDR), Ceramide synthase (CerS) and dihydroceramide desaturase (DEGS) are represented by their acronyms.

All the reactions implicated in *de novo* pathway occur in the endoplasmic reticulum (ER) (Figure 2). The pathway begins with the condensation of palmitate and serine to give rise 3-keto-dihydrosphingosine (also called 3-ketosphinganine) by serine palmitoyltransferase (SPT), a rate-limiting enzyme for *de novo* pathway. The SPT is a complex formed by SPTLC1, SPTLC2, ssSPTa/b and a negative regulatory subunit ORMDLs (homologs of the yeast and plant Orms) (Davis et al., 2019; Wattenberg, 2021). It should be noted that sphingolipids can be made up of chains of different lengths. In this first reaction, ssSPTa/b subunits increase enzyme activity and the specificity for the acyl-CoA substrate. Interestingly, mutations in ssSPTa/b that result in elevated 18-carbon (C_18_-) sphingoid long-chain bases stimulate neurodegeneration (Zhao et al., 2015). Then, the enzyme 3-keto-dihydrosphingosine reductase (KDR) catalyzes the reduction of 3-ketosphinganine to sphinganine. Later, ceramide synthase (CerS) leads to the incorporation of another acyl-CoA to turn the sphinganine into dihydroceramide (dhCer). To date, six isoforms of CerS (known as CerS1-6) have been discovered in mammals and plants (Kim J. L. et al., 2021). The main difference between those isoforms is their affinity for acyl-CoA molecules depending on the length of the chain. Thusly, CerS1 mainly produces 18 carbon chain Cer (C_18_-Cer), CerS2 generates to C_22/24_-Cer, CerS3 give rise C_26_-Cer, CerS4 synthesizes C_18/20_-Cer, CerS5 forms C_14/16_-Cer and CerS6 produces C_14/16_-Cer. Interestingly, CerS1 is highly expressed in cells belonging to the nervous system. The last step implicates an addition of a double bond in position 4-5 trans of dhCer by the activity of dihydroceramide desaturase (DEGS).

#### Serine Palmitoyltransferase

It is well established that SPT is the main regulator of the *de novo* pathway. Overexpression of SPT increases Cer levels and consequently enhances apoptosis, cellular oxidative stress, and mitochondrial fission (Granado et al., 2009a; Bekhite et al., 2021).

Transient coronary occlusion studies in mice, a model of myocardial infarct, demonstrated an elevation in Cer levels after reperfusion, correlated with high levels in pro-inflammatory cytokines, such as IL-6, IL-1β or TNF-⍺, as well as an increase in ROS in ischemic tissues (Reforgiato et al., 2016). Interestingly, an increase in the levels of Cer was correlated with higher expression of SPTLC1 and 2. Furthermore, the intraventricular administration of myriocin, a specific inhibitor of SPT, reduced the area of myocardial ischemic damage, reducing the levels of Cer and pro-inflammatory molecules (Reforgiato et al., 2016). However, these studies have not been transferred to the cerebral ischemia model; representing an interesting target to study deeper. In addition, cerebral microendothelial hypoxia induces elevated levels of dhCer, and contradictorily, myriocin promoted endothelial barrier dysfunction and caspase-3 expression in response to oxygen deprivation (Testai et al., 2014). It is well determined that hypoxia stimulates neuronal apoptosis and cell death, by upregulation of SPT (Kang et al., 2010) or its aggregation (Dedov et al., 2004), respectively.

Vascular tone is a determining factor for the regulation of blood pressure. High values of blood pressure (hypertension) are a well-known risk factor for stroke (Wajngarten and Silva, 2019). Of interest, plasma Cer levels are increased in hypertension (Spijkers et al., 2011; Mielke et al., 2019). However, it has recently been observed that in stroke-prone hypertension model rats the expression of SPTLC1 and SPTLC2 are decreased in the brain compared to wild-type rats (Pepe et al., 2021). Moreover, the depletion of SPTLC2 in mice showed that C_16:o_-, C_24:0_-, and C_24:1_-Cer were observed essentials in vascular and blood pressure homeostasis, and establish the endothelium as a crucial source of plasma Cer (Cantalupo et al., 2020). Accordingly, disruption of sphingolipid metabolism, with an increase in SPT activity, augments Cer-induced autophagy in preeclampsia (Melland-Smith et al., 2015). In this regard, sphingolipid profiling of human fetoplacental vasculature in preeclampsia demonstrated high levels of Cer and dhCer, due to an upregulation of SPT, which was reversed by Nogo-B activation (del Gaudio et al., 2020). Nogo-B protein (also called reticulon-4B) is ubiquitously expressed in peripheral tissues, being implicated in several functions such as inflammation or vasculature remodeling. Interestingly, previous works reported that mice lacking Nogo-B were protected against endothelial dysfunction and cardiac failure (Zhang et al., 2016; Cantalupo et al., 2020). Additionally, previous studies demonstrated that Cer promotes endothelial dysfunction through the inhibition of eNOS (Li et al., 2002; Xing et al., 2006), being reversed by the inhibition of SPT by myriocin (Zhang et al., 2012).

Interestingly, myocardial SPTLC1 and SPTLC2 levels were also found to increase in heart failure patients (Hoffman et al., 2021). Cardiomyocyte Krüppel-Like Factor 5 (KLF5) a transcription factor implicated in fatty acid oxidation was induced during the development of ischemic heart failure and stimulated SPT expression. Moreover, KLF5 ablation suppressed SPT expression, contributing to eccentric remodeling in ischemic cardiomyopathy (Hoffman et al., 2021).

As described above, pro-inflammatory cytokines and caspase-3 are directly involved in the evolution of ischemic lesions. Concerning pro-inflammatory cytokines, a recent work has described that inhibition of SPT with ARN14494 prevents the synthesis of pro-inflammatory cytokines (TNF-α and IL-1β), growth factor TGF-β1, and oxidative stress-related enzymes iNOS and COX2 in mouse primary cortical astrocytes (de Vita et al., 2019). Furthermore, ARN14494 showed to be neuroprotective in primary cortical neurons, decreasing neuronal death and caspase-3 activation (de Vita et al., 2019).

#### 3-Keto-Dihydrosphingosine Reductase

As previously stated, KDR catalyzes the reduction of 3-ketosphinganine to sphinganine. Recently, sphinganine levels were observed to decrease hours after transient middle cerebral artery occlusion (tMCAO) in rats and mice with a correlation with an increase in Cer levels (Yang Y. et al., 2018; Chao et al., 2019). Curiously, treatment with a well-established neuroprotectant such as isosteviol sodium or, atorvastatin, a drug in the statin family to lower cholesterol, significantly increased sphinganine levels (Yang Y. et al., 2018; Chao et al., 2019). These data could indicate a possible implication of KDR activity or up-regulation of CerS in the evolution of ischemic lesions; therefore, more investigations should be addressed. Interestingly, recent work has demonstrated a reduction of KDR expression in spontaneously hypertensive rat stroke-resistance in the brain (Pepe et al., 2021).

#### Ceramide Synthase

As described above, different isoforms of CerS give rise to Cer of different chains. Studies in tMCAO models have shown that there is an increase in levels of long and very-long-chain Cer in plasma following reperfusion (Yang Y. et al., 2018; Chao et al., 2019). In fact, recent studies have described similar results in patients after a stroke (Gui et al., 2020; Lee et al., 2021).

As mentioned, there is a decrease in sphinganine levels in tMCAO models (Yang Y. et al., 2018; Chao et al., 2019). Sphinganine can be phosphorylated by sphingosine kinase to produce S1P. Interestingly, a decrease in S1P levels has been observed in rats after tMCAO (Wu et al., 2020). In this regard, the treatment with nuciferine, a compound that has been shown to be effective against cerebrovascular diseases, increased S1P levels (Wu et al., 2020). A decrease in CerS activity could lead to the accumulation of sphinganine and thus an increase in sphinganine 1-phosphate, being an interesting target for further studies.

Cer species with diverse acyl chain lengths have been detected in the mitochondria from brain tissue (Novgorodov and Gudz, 2011; Mignard et al., 2020). Likewise, CerS1, CerS2 and CerS6 enzymes can be found in the mitochondrial membrane, inducing the synthesis of C_18_-, C_22_- and C_16-_Cer, respectively (Yu et al., 2007). SIRT-3 is a member of an evolutionarily conserved family of NAD^+^-dependent deacetylase and mono-ADP-ribosyltransferase implicated in the regulation of metabolism and mitochondrial homeostasis. Interestingly, deacetylase sirtuin-3 (SIRT3)-null mice showed an increase in the acetylation of CerS1, 2 and 6, and the consequent reduction in Cer production, promoting mitochondrial protection and neuroprotective effect on the MCAO model (Novgorodov S. S. A. et al., 2016). However, there are controversies about the neuroprotective capacity of SIRT-3, possibly due to the different experimental models of stroke used in each study and, according to the study, mitochondrial or endothelial dysfunction (Verma et al., 2018; Yang X. et al., 2018, 2021). Similarly, mitochondrial CerS activity was associated with mitochondrial injury in cerebral ischemia/reperfusion, and was abolished in c-Jun N-terminal kinase-3 (JNK3)-deficient mice, suggesting a pivotal role of JNK-3 in the regulation of Cer biosynthesis in cerebral ischemia (Yu et al., 2007). Dynamin-related protein (Drp-1) is a protein involved in the mitochondrial biogenesis and maintenance of healthy mitochondria (Smirnova et al., 2001). Recently, it was observed the implication of Drp-1 in mitochondrial dysfunction during ischemic injury (Duan et al., 2020). Interestingly, the deletion of neuron-specific Bβ2 regulatory subunit of protein phosphatase 2A (PP2A), an activator of Drp-1 protects against cerebral ischemia (Flippo et al., 2020). In addition, Drp-1 modifies Cer distribution in the outer membrane of the mitochondria, preventing mitophagy (Fugio et al., 2020). Furthermore, it was observed that TNF-α increases intracellular levels of Cer, leading to an increase in the activity of PP2A and a pro-inflammatory cascade in lung epithelial cells (Cornell et al., 2009).

Of interest, recent work demonstrated elevated mRNA levels of CerS3 and 6 in brain endothelial cells of evoked autoimmune encephalomyelitis mice, suggesting their implication in the rising levels of Cer related to endothelial dysfunction (Schmitz et al., 2021).

#### Dihydroceramide Desaturase (DEGS or Des)

It is well established that dhCer stimulates cell cycle arrest and apoptosis (Ordóñez et al., 2016b; Casasampere et al., 2016; Casasampere et al., 2017), and recently it was also demonstrated that dhCer induces endothelial impairment (Savira et al., 2021). Oxidative stress is one of the main problems due to mitochondrial dysfunction. Studies in different cell lines have shown that the stimulation of oxidative stress by H_2_O_2_ leads to dhCer accumulation, indicating inhibition of DEGS that is directly related to oxidative stress (Idkowiak-Baldys et al., 2010). Furthermore, DEGS mutation (*DEGS1* ^*H132R*^) resulting in gene inactivation provokes mislocalization of Rac1 to the endolysosomes, where it forms the NOX complex with NADPH-oxidase, promoting cytosolic ROS generation (Tzou et al., 2021). In addition, hypoxic conditions lead to apoptosis-mediated dhCer accumulation due to an inhibition of DEGS. Moreover, overexpression of DEGS reverted cell death and stimulated cell proliferation upon hypoxia (Devlin et al., 2011).

As mentioned above, obesity is a risk factor to suffer stroke. *In vitro* and *in vivo* experiments with *desg1* ^+/-^ mice demonstrated that a reduction of DEGS expression prevents Cer accumulation, impaired eNOS phosphorylation, and endothelial dysfunction in high-fat diet models (Zhang et al., 2012).

Supporting this evidence about the implication of DEGS in stroke, recent preclinical studies detected an elevation of dhCer levels in mouse brains 24 h after tMCAO in the ipsilateral hemisphere (Chao et al., 2019).

### The Sphingomyelinase Pathway

**FIGURE 3:**
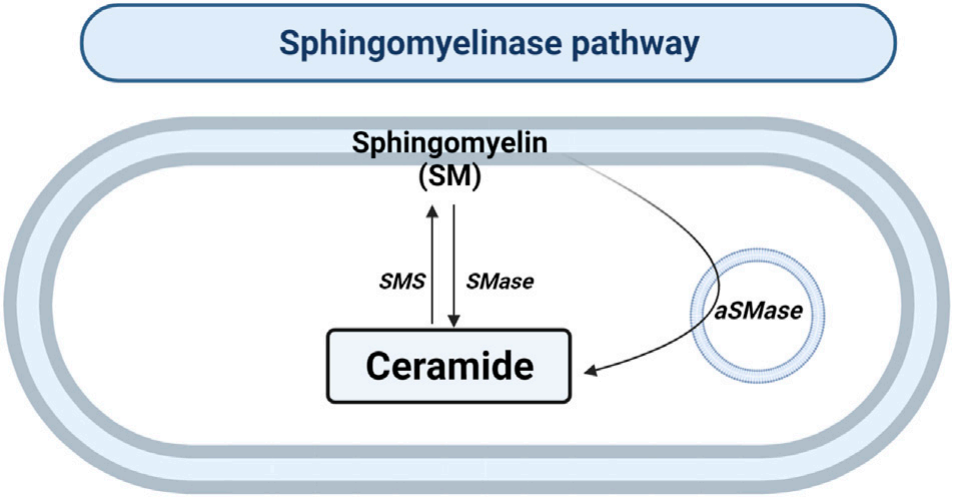
Sphingomyelinase pathway. Solid arrows represent single reactions. Sphingomyelinase (SMase), acid sphingomyelinase (aSMase) and sphingomyelin synthase (SMS) are represented by their acronyms.

SMase hydrolyzes the sphingomyelin (SM) located in the plasma membrane to generate Cer and phosphocholine in the cytosol or lysosomes (Figure 3). To date, there are five types of SMases described. SMases can be classified according to ionic regulation, location, and optimal pH of activity. Therefore, the acid SMase (aSMase) (Gorelik et al., 2016) can be detected in the lysosomes and plasma membrane (Gorelik et al., 2016); neutral Mg^2+^-dependent and neutral Mg^2+^-independent SMase (nSMase) have been found in ER, nucleus and plasma membrane (Clarke et al., 2006); alkaline SMase (alkSMase) has been observed in intestinal tract lumen and human bile (Goni and Alonso, 2002; Cataldi et al., 2020); and Zn^2+^-dependent secreted form of aSMase involved in the degradation of SM obtained in the diet (Kornhuber et al., 2015). Oppositely, sphingomyelin synthase (SMS) catalyzes the production of SM from Cer. So far, three different SMSs were described; SMS1, SMS2 and SMS-related protein (SMSr) (Chen and Cao, 2017).

#### Sphingomyelinase

The SMase pathway is able to increase the intracellular Cer pool quickly. aSMase and nSMase can be activated by pro-inflammatory molecules such as TNF-α, lipopolysaccharide (LPS), IL-1β, IL-6, or cytosolic phospholipase A2 (cPLA_2_), giving rise to an elevation of intracellular Cer concentrations (Malaplate-Armand et al., 2006; Becker et al., 2010; Jenkins et al., 2010; Gomez-Muñoz et al., 2013; Lallemand et al., 2018). Of interest, there are three isoforms of nSMase; where nSMase1 is mainly expressed in the kidney, nSMase is mostly detected in brain tissue, and nSMase3 is ubiquitously expressed in all cell types (Clarke et al., 2006, 2011).

After tMCAO in the mouse model, the activity of the aSMase was increased with an elevation of Cer and inflammatory cytokines (Yu et al., 2000). However, mice lacking aSMase (*smpd1* ^*−/−*^) revealed a reduction in the infarct volume and an improvement of the behavioral outcome, with a decrease in Cer and pro-inflammatory molecules (Yu et al., 2000). However, recent work demonstrated that in the mouse model of homozygous deficiency of aSMase (*smpd1* ^*−/−*^) there is an aggravation of brain ischemia/reperfusion injury due to an increase in blood-brain barrier permeabilization and leukocyte infiltration by overexpression of I-CAM1 molecules (Hagemann et al., 2020). Nonetheless heterozygous mice (*smpd1* ^*+/-*^) were protected against MCAO (Hagemann et al., 2020).

The term FIASMA (Functional Inhibitor of Acid sphingomyelinase) was proposed to encompass a family of drugs, many of them approved as antidepressants for use in humans, which have been shown to inhibit the aSMase activity (Kornhuber et al., 2010). Currently, several FIASMA molecules have been investigated in ischemic stroke demonstrating a neuroprotective role, such as fluoxentine (Mohamud Yusuf et al., 2019). However, studies with FIASMAs must be deepened since there are controversies about the neuroprotective capacity depending on the pathology of certain compounds (Brunkhorst et al., 2015; van der Worp, 2019). In addition, as observed in this review, the diversity of pathways that affect the production of Cer must be addressed in the studies (Brunkhorst et al., 2015).

Recently it has been developed a small molecule, called ARC39, that was able to inhibit both isoforms of aSMase *in vitro* (Naser et al., 2020). However, the *in vivo* ARC39 application was not be confirmed yet; therefore, more studies should determine the effectivity of ARC39 *in vivo*. Furthermore, a potent light-inducible compound, called PhotoCaged ASM Inhibitor (PCAI), has been developed (Prause et al., 2020). Interestingly, PCAI can be directed to damaged tissue, such as the ischemic brain.

Deregulation of nSMase activity has been observed in Alzheimer’s disease, Parkinson’s disease, cognitive dysfunction and cerebral ischemia (Tabatadze et al., 2010; Wu et al., 2010; Gu et al., 2013; Shamseddine et al., 2015; Cataldi et al., 2017; Custodia et al., 2021). Altura *et al* studied the effects in the brain of the nSMase, as well as Cer analogs, phosphorylcholine and Cer metabolites (Altura et al., 2002). This work demonstrated that nSMase and Cer could induce cerebrovascular damage by vasospasm, vascular constriction, chemoattraction of leukocytes and increased BBB permeability (Altura et al., 2002). Moreover, antioxidants such as vitamin E, protein kinase C-⍺ (PKCα) inhibitor (Gö6976), MAP kinase inhibitor (PD98059), and Ca^2+^ channel blockers (nimodipine) shown to decrease nSMase activity and could reduce cerebrovascular damage (Altura et al., 2002). nSMase, mainly nSMase2 plays an important role in the generation of Cer in hippocampal astrocytes of rats. A1B adenosine receptor participates in the increment of nSMase2 induced by p38MAPK phosphorylation and the accumulation of Cer during cerebral ischemia. The inhibition of nSMase2 by GW4869 was found to reduce the levels of IL-1β, IL-6 and TNF-α in hippocampal neuronal culture in hypoxia conditions (Gu et al., 2013).

Sackmann et al. demonstrated the reduction of nSMase2, dhCer, SM and Cer levels in neurons undergoing hypoxia, as a model of oxidative stress. However, the inhibition or deletion of nSMase2 using siRNA or knock-out, respectively, was not enough to reduce Cer and SM levels (Sackmann et al., 2019).

#### Sphingomyelin Synthase

It was demonstrated that the selective inhibition of SMS by tricyclodecan-9-yl-xanthogenate (D609) induces an increase in the concentration of Cer in the ER, triggering autophagy in hippocampal neurons (Gulbins et al., 2018).

Transcriptional studies revealed a reduction in SMS1 levels at 3 and 24 h in the ipsilateral cortex compared to the contralateral one after the occlusion in tMCAO rat models (Dmitrieva et al., 2008). Interestingly, a reduction in the subcortex was observed in all groups, possibly due to surgery.

SMS2 knockout (SMS2^−/−^) mice showed a significant improvement of cognitive function and minimized infarct volume at 72 h after tMCAO (Xue et al., 2019). The underlying mechanism seems to be the impairment of SM in the membrane that would lead to the reduction of the TLR4 complex in the microglial membrane. Likewise, SMS2 deficiency was shown to inactivate the NF-κB pathway in HEK-293 cells. Recently, it was shown that the lack of SMS2 also suppressed the activation of microglia through the inhibition of NF-κB and reduced ischemic injury. (Yang et al., 2020).

Of interest, high levels of apolipoprotein A-I in blood, the main component of high-density lipoproteins (HDL) and cholesterol, are at a high risk of suffering from vascular diseases. In this regard, it has been described that the overexpression of SMS1 and SMS2 stimulates the intracellular accumulation of cholesterol in the human hepatoma cell line (Yan et al., 2011). Furthermore, the adenoviral-mediated overexpression of SMS1 and SMS2 lead to an increase of both the atherogenic potential and the apolipoprotein B in mice (Dong et al., 2006).

### The Salvage Pathway

**FIGURE 4:**
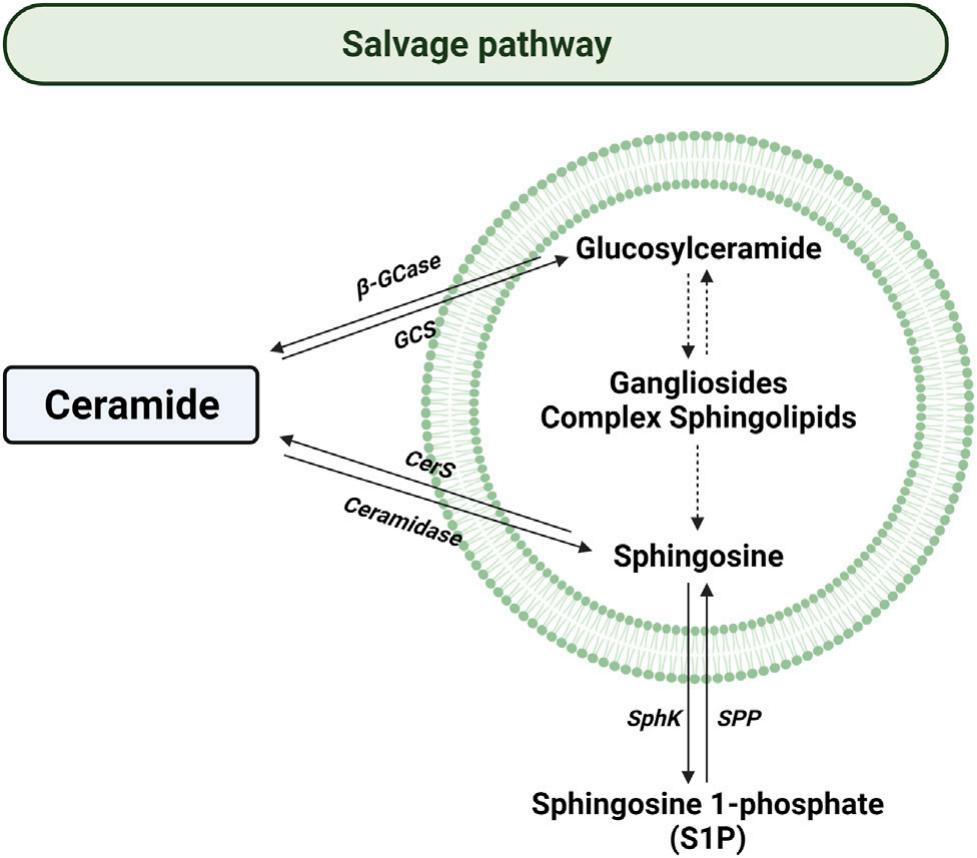
Salvage pathway. Solid arrows represent single reactions, whereas dashed arrows represent various step reactions. Acid β-glucosidase (β-GCase), glucosylceramide synthase (GCS), ceramide synthase (CerS), sphingosine kinase (SphK), and sphingosine 1-phosphate phosphatase (SPP) are represented by their acronyms. The lipid bilayer represents lysosomes.

The salvage pathway consists of a series of catabolic reactions driving to complex sphingolipids degradation in lysosomes (Figure 4). The pathway begins with the degradation of complex sphingolipids to give rise to lactosylceramide (LacCer) by different reactions. The opposite reaction is due to lactosilceramide synthase. Then, LacCer is hydrolyzed to produce glucosylceramide (GluCer) by the activity of LacCer hydrolase. After, acid β-glucosidase 1 (β-GCase) hydrolyzes the GluCer to form Cer. Contrary, glucosylceramide synthase (GCS) converts Cer into GluCer. Later the Cer is transformed to sphingosine by the acidic ceramidase (ASAH1) in order to be transported to the cytosol (Vijayan et al., 2019).

In the cytosol, sphingosine can be converted back to Cer by the action of ceramide synthase. This balance can be reverted by the action of cytosolic ceramidase that degrade Cer to produce sphingosine. So far, different ceramidases have been described, such as alkaline ceramidases (encoded by ACER1, ACER2, and ACER3 genes), the previously named ASAH1, and neutral ceramidase (ASAH2) (Romiti et al., 2000; Gebai et al., 2018; Coant and Hannun, 2019; Xu et al., 2021). Meanwhile, ASAH1 is ubiquitously located in lysosomal compartments; ASAH2 is mainly expressed in the plasma membrane of the small intestine and colon (Coant and Hannun, 2019).

#### Lactosylceramide Synthase

Several studies have demonstrated that the accumulation of LacCer induces apoptosis (Martin et al., 2006; Bodas et al., 2015), mitochondrial dysfunction (Novgorodov S. A. et al., 2016) and IL-6 release (Bodas et al., 2015). Likewise, it was reported the increment of LacCer in atherosclerotic plaques (Edsfeldt et al., 2016).

Interestingly, it was found that the accumulation of LacCer promoted the migration of neutrophils to the endothelium through the stimulation of CD11/CD18 integrins (Mac-1) and ICAM-1, respectively; by a mechanism dependent on cytosolic phospholipase 2 (cPLA_2_) (Arai et al., 1998; Bhunia et al., 1998). Furthermore, LacCer stimulated the generation of ROS. Later, it was reported that stimulation with TNF-⍺ promotes the accumulation of LacCer, promoting the phosphorylation of cPLA_2_ and generation of ROS (Nakamura et al., 2017). In this sense, LacCer was also described to recruit protein kinase C (PKC) and cPLA_2_ to stimulate PECAM-1 expression in human monocytes, and subsequent their adhesion to endothelial cells (Gong et al., 2004). Interestingly, gene silencing by siRNA and chemical blocking with specific inhibitors of LacCer synthase succeed to inhibit the expression of VEGF receptor and, consequently, angiogenesis in human umbilical vein endothelial cells huvec (Rajesh et al., 2005).

LacCer was reported to induce cell migration *in vitro* by stimulating extracellular signal-regulated kinases (ERK1/2) (Mu et al., 2009). In addition, LacCer stimulated membrane proteins involved in circulating cell recruitment, such as platelet-derived growth factor receptor beta (PDGFR-β) or integrins (αv and β3); and MMP-1 and MMP-2 production (Mu et al., 2009). Interestingly, the implication of LacCer in monocyte migration was recently corroborated in patients with acute myocardial infarction by plasma lipidomics analysis (Fiorelli et al., 2021).

#### Acid β-glucosidase

It is well known that mutations in the acid β-glucosidase gene (*GBA*) lead to the development of Gaucher’s disease (Hruska et al., 2008). Therefore, most of the studies have been directed to Gaucher’s disease. However, ambroxol a mucolytic drug used to treat Gaucher’s and Parkinson’s disease in a clinical trial (Istaiti et al., 2021), showed encouraging data for the treatment of ischemia. Post-stroke pneumonia is a common complication in patients that suffered cerebrovascular ischemia (Li et al., 2019). Interestingly, patients treated with ambroxol obtained a better recovery after ischemic stroke or intracerebral hemorrhage (Jiang et al., 2020; Istaiti et al., 2021). Recently, ambroxol was described to upregulate acid β-glucosidase and promote neural stem cells differentiation through activation of Wnt/β-Catenin pathway (Ge et al., 2021). Also of interest, intravenous infusion of induced pluripotent stem cells (iPSC)-derived neural precursor cells in Gaucher’s disease mouse model increased the brain acid β-glucosidase activity, showing an improvement in sensorimotor function and a prolonged life span (Peng et al., 2019).

#### Glucosylceramide Synthase

The vast majority of studies on this pathway emphasize that the accumulation of GluCer and thus the decrease in Cer levels is neuroprotective, since glycosylation of Cer constitutes a protective process for the cell by reducing Cer levels (Bleicher and Cabot, 2002). However, it is well established that high concentrations of GluCer also promote apoptosis and necrosis (Edsfeldt et al., 2016).

There was reported a decrease of GCS activity in the ischemic tissue from spontaneously hypertensive rats undergoing MCAO injury (Takahashi et al., 2004). Preconditioning strategies have been observed as neuroprotector against ischemia/reperfusion injury. Interesntingly, preconditioning strategy in rats showed a neuroprotection in ischemic conditions by activation of GCS and subsequent reduction of intracellular ceramide accumulation after pMCAO in the first hours (Takahashi et al., 2004). In addition, neurons upon hypoxia condition also showed elevated levels of GluCer due to an increase in Cer levels (Kang et al., 2010). The specific inhibition of GCS increased the ratio of cell death, indicating that the increase in GluCer has a protective role by decreasing the levels of Cer (Kang et al., 2010). Cerebral micro-endothelial cells subjected to hypoxic conditions showed an increased GCS activity. Additionally, GCS inhibition by EtPoD4 induced endothelial barrier dysfunction and caspase-3 upregulation (Testai et al., 2014). Furthermore, GluCer was detected at high concentration in human atherosclerotic plaques, which was associated with pro-inflammation (Edsfeldt et al., 2016). *In vitro* experiments with human coronary artery smooth muscle cells (HCASMCs) revealed that GluCer treatment stimulated pro-inflammatory molecules releases, such as IL-6, TNF-⍺, monocyte chemoattractant protein-1 (MCP-1), macrophage inflammatory protein-1β (MIP-1β), and chemokine (C-C motif) ligand 5 (CCL5, also known as RANTES) (Edsfeldt et al., 2016). Interestingly, a study showed that a high GluCer diet reduced memory impairment in aged mice, correlated with a decrease in the levels of iNOS, COX-2, IL-1β and TNF-⍺ (Lee et al., 2013).

#### Ceramidases

The ceramidase activity has two functions; 1) to stimulate the production of Cer by participating in the degradation of complex sphingolipids in the lysosome, and the opposite one, 2) to reduce the levels of Cer in the cytosol by increasing the levels of sphingosine (Parveen et al., 2019). Sphingosine can be subsequently phosphorylated by sphingosine kinase to produce sphingosine 1-phosphate. Sphingosine 1-phosphate is the most studied sphingolipid, being involved in several functions such as proliferation, migration, inflammation and vasodilation, among others (Watterson et al., 2003; Roviezzo et al., 2011; Czubowicz et al., 2019).

It is well established that ceramidases can be secreted by endothelial cells, macrophages and fibroblasts, being able to participate in the extracellular metabolism of sphingolipids, highly related to atherogenesis (Romiti et al., 2000). Interestingly, it has been reported that *ASAH1* deficient mice display central system nervous abnormalities (Sikora et al., 2017); along with being, associated with Alzheimer’s disease, diabetes and cancer (Gebai et al., 2018).

To date, although the implication of ceramidases in the pathogenesis of cerebral ischemia has not been addressed. However, there is evidence about its possible involvement. As mentioned above, several antidepressants have been studied for their application in the treatment of cerebral ischemia. For example, desipramine is an antidepressant that is able to inhibit ASAH1 (Elojeimy et al., 2006), along with being neuroprotective in the treatment of transient global ischemia observed in mice upon bilateral common carotid artery occlusion (BCCAO) (Gaur and Kumar, 2010).

Autophagy is a vital process for the maintenance of cellular homeostasis. In recent years it has been shown that autophagy could act as a neuroprotective strategy in ischemia (Nabavi et al., 2019). It is well established that activation of unc-51 Like Autophagy Activating kinase 1 (Ulk1) by its phosphorylation at residue Ser 555 induces autophagy (Kim et al., 2011). Recently, it was demonstrated that JLX-001 compound showed neuroprotective properties in stroke via AMPK-Ulk1 (Aoyao et al., 2019). Fascinatingly, overexpression of *ASAH1* in human aortic endothelial cells (HAECs) stimulated phosphorylation of Ulk-1, independently of adenine monophosphate-activated protein kinase AMPK (Weikel et al., 2015).

Different studies have observed the involvement of exosomes in the evolution of stroke through the transport of proteins, lipids and genetic materials between cells (Zhang and Chopp, 2016). Recently, it has been determined that *ASAH1* ^*−/−*^ mice stimulated the accumulation of exosomes in the endothelium of small coronary arteries (Yuan et al., 2021). This process is related to coronary microvascular dysfunction (CMD), which is one of the major factors contributing to ischemic heart disease.

High-mobility group box1 protein (HMGB1) is a novel high conserved cytokine-like protein ubiquitously expressed that is involved in numerous inflammatory processes. HMGB1 was found to mediate cerebral inflammation and brain injury through its interaction with toll-like receptors (TLRs), MMP, and receptor for advanced glycation endproducts (RAGE) during stroke; promoting pro-inflammation and BBB breakdown (Ye et al., 2019). Recently, it has been reported that HMGB1 induces migration and proliferation of mouse coronary arterial myocytes and endothelial progenitor cells (Zhang BCE et al., 2019; Yuan et al., 2020), in addition to downregulation of *ASAH1* and Cer accumulation (Yuan et al., 2020).

**FIGURE 5:**
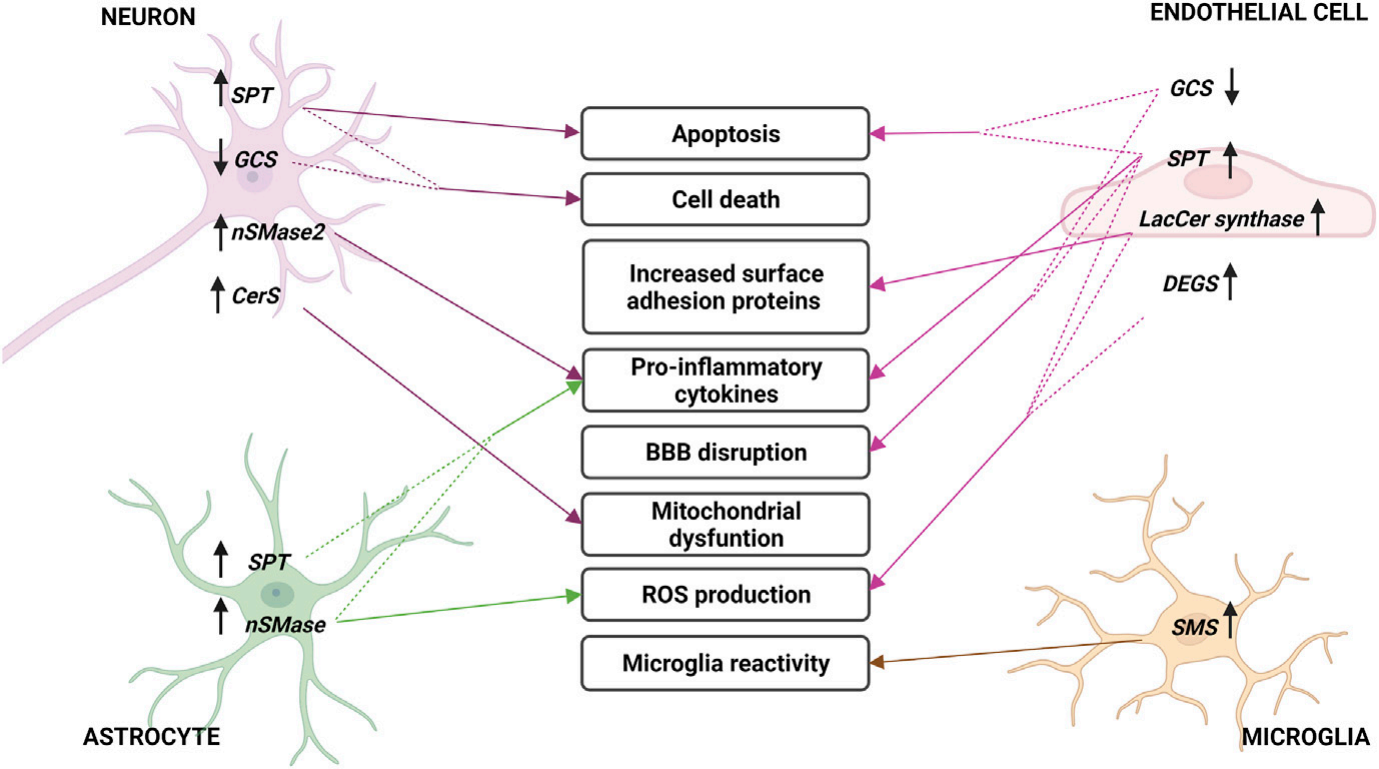
Sphingolipid metabolism enzymes involved in cerebral ischemia. Changes in sphingolipid enzymes in different cell types of brain parenchyma and endothelium during stroke. In the middle, the consequences of these variations are described, indicated with arrows. Arrows next to enzymes describe an increase (up) or decrease (down) in their expression or activity. Acronyms represent: serine palmitoyltransferase (SPT), neutral sphingomyelinase (nSMase), Lactosyl-ceramide synthase (LacCer synthase), dihydroceramide desaturase (DEGS), sphingomyelin synthase (SMS), glucosylceramide synthase (GCS) and ceramide synthase (CerS).

Figure 5 and table 1 summarize all the points discussed in the review related to ceramide metabolism and its implication with the develop and progression of stroke.

**TABLE 1:** shows a schematic summary of how certain manipulations in ceramide metabolism affect direct or undirectly ischemia/hypoxia events that have been discussed in this review.

Target	Molecule/Model	Consequence	Model	Reference
SPT	Myriocin	SPT inhibition - Reduction of myocardial reperfusion injury	Left anterior descending (LAD) coronary ligature mouse model	Reforgiato et al. (2016)
		SPT inhibition - Induction of endothelial barrier dysfunction and increased caspase-3 levels	HCEC cell line in oxygen deprivation	Testai et al. (2014)
		SPT inhibition - Reversion of eNOS inhibition-mediated endothelial dysfunction	Diet-induced obesity mouse model	Zhang et al. (2012)
	KLF5 ablation	KLF5 Knock-out SPT downregulation - Prevention of ceramide accumulation and alleviation of eccentric remodeling	Myocardial infarction mouse model	Hoffman et al. (2021)
	ML264	KLF5 inhibition. SPT downregulation - Prevention of ceramide accumulation and alleviation of eccentric remodeling		
	ARN14494	SPT inhibition - Reduction in the release of pro-inflammatory cytokines and caspase-3 production in astrocytes and neurons, respectively	Primary neuronal culture exposed to β-amyloid	de Vita et al. (2019)
CerS	SIRT3-null mice	Acetylation of CerS and Cer levels reduction—Mitochondrial protection and neuroprotection	MCAO mouse model	Novgorodov et al. (2016b)
	JNK3-deficient mice	Ceramide synthase inhibition—mithochondrial protection		Yu et al. (2007)
DEGS	*DEGS* mutant gene	DEGS downregulation—Increased cytosolic ROS generation	SH-SY5Y cells	Tzou et al. (2021)
	DEGS transfection	DEGS overexpression—Stimulation of cell proliferation and rescue from apoptosis	MCF-7, MDA231, and 468 human breast cancer cell lines upon hypoxia	Devlin et al. (2011)
SMase	*smpd* ^*−/−*^ mice	Lack of aSMase—Reduction of infarct volume and an improvement of behavioral outcome	tMCAO mouse model	Yu et al. (2000)
	*smpd* ^*−/−*^ mice	Lack of aSMase—Increment of blood-brain barrier permeabilization and leukocyte infiltration	MCAO mouse model	Hagemann et al. (2020)
	*smpd* ^*+/-*^ mice	Lack of aSMase—Reduction in ischemia/reperfusion injury		
	Fluoxetine	SMase inhibition—Neuroprotective effects	MCAO mouse model and neuronal culture	(Kim et al., 2007; Lim et al., 2009)
	Fluoxetine	SMase inhibition—No effects	Clinical trial	van der Worp, (2019)
	Antioxidant treatment	nSMase activity decreased—Reduction of vascular constriction, leukocyte infiltration and BBB permeability	Rat	Altura et al. (2002)
	GW4869	nSMase activity decreased -Reduction of the levels of IL-1β, IL-6 and TNF-α	Rat hippocampal neurons	Gu et al. (2013)
SMS	D609	SMS inhibition—Induction of autophagy due to an elevation in Cer concentration	Hippocampal neurons	Gulbins et al. (2018)
	*SMS2* ^*−/−*^	SMS2 decrease—improvement of cognitive function	*SMS* ^*−/−*^ mouse model tMCAO	Xue et al. (2019)
	*SMS2* ^*−/−*^	SMS2 decrease—suppression of glia activation and improvement of cognitive function	*SMS* ^*−/−*^ mouse model tMCAO	Yang et al. (2020)
GCS	Preconditioning strategies	Increment of GCS activity - neuroprotection	Rat pMCAO model	Takahashi et al. (2004)
	EtPoD4	GCS inhibition - endothelial barrier dysfunction and caspase-3 upregulation	Cerebral micro-endothelial cells upon hypoxia conditions	Testai et al. (2014)
Ceramidase	Desipramine	ASAH1 inhibition—neuroprotective effect	BCCAO mouse model	Gaur and Kumar, (2010)

## Concluding Remarks

There is a large body of evidence linking sphingolipids to the progression and severity of ischemic stroke. Ischemic brain damage involves different cell types, etiologies, signaling and stress processes, which is why it is so complex to study. Furthermore, taking into account that sphingolipid metabolism is ubiquitously present in all cell types with involvement in different processes, they make their study difficult to interpret. However, several preclinical studies have shown that the manipulation of its metabolism exerts neuroprotective functions. Recent studies, both preclinical and clinical, of mass spectrometry to detect different species of sphingolipids in plasma have corroborated the implication of sphingolipids on the severity of stroke. So far, sphingolipidomics requires a series of processing steps and an investment that makes its analysis expensive. In addition, not all sphingolipids can be detected in the same analysis, requiring several. Therefore, the development of new techniques to measure sphingolipids is critical to analyzing all the parameters in all medical facilities. In addition, the development of chemical compounds that alter the activity of these enzymes is of vital importance for the development of viable and effective drugs. For this reason, in-depth studies are required to elucidate the specific role of sphingolipids in the acute damage and subsequent neurorepair processes associated to ischemic stroke.
